# TSPAN6 reinforces the malignant progression of glioblastoma via interacting with CDK5RAP3 and regulating STAT3 signaling pathway

**DOI:** 10.7150/ijbs.85984

**Published:** 2024-04-15

**Authors:** Chong Zhang, Fei-hua Du, Rou-xin Wang, Wen-bo Han, Xing Lv, Ling-hui Zeng, Guo-qing Chen

**Affiliations:** 1 Affiliated Luqiao Hospital, School of Medicine, Hangzhou City University, Hangzhou, Zhejiang, China, 310015.; 2 School of Medicine, Hangzhou City University, Hangzhou, Zhejiang, China, 310015.; 3 Key Laboratory of Novel Targets and Drug Study for Neural Repair of Zhejiang Province, School of Medicine, Hangzhou City University, Hangzhou, Zhejiang, China, 310015.; 4 Department of Pharmacology, Zhejiang University, Hangzhou, Zhejiang, China, 310058.

**Keywords:** TSPAN6, glioblastoma, angiogenesis, STAT3, CDK5RAP3

## Abstract

Glioblastoma is the prevailing and highly malignant form of primary brain neoplasm with poor prognosis. Exosomes derived from glioblastoma cells act a vital role in malignant progression via regulating tumor microenvironment (TME), exosomal tetraspanin protein family members (TSPANs) are important actors of cell communication in TME. Among all the TSPANs, TSPAN6 exhibited predominantly higher expression levels in comparison to normal tissues. Meanwhile, glioblastoma patients with high level of TSPAN6 had shorter overall survival compared with low level of TSPAN6. Furthermore, TSPAN6 promoted the malignant progression of glioblastoma via promoting the proliferation and metastatic potential of glioblastoma cells. More interestingly, TSPAN6 overexpression in glioblastoma cells promoted the migration of vascular endothelial cell, and exosome secretion inhibitor reversed the migrative ability of vascular endothelial cells enhanced by TSPAN6 overexpressing glioblastoma cells, indicating that TSPAN6 might reinforce angiogenesis via exosomes in TME. Mechanistically, TSPAN6 enhanced the malignant progression of glioblastoma by interacting with CDK5RAP3 and regulating STAT3 signaling pathway. In addition, TSPAN6 overexpression in glioblastoma cells enhanced angiogenesis via regulating TME and STAT3 signaling pathway. Collectively, TSPAN6 has the potential to serve as both a therapeutic target and a prognostic biomarker for the treatment of glioblastoma.

## Introduction

The glioma is a common form of malignant brain tumor characterized by its diffuse infiltration, lack of distinct boundaries, uncontrolled proliferation, and significant invasiveness 1. Glioblastoma, classified as a grade IV glioma by the World Health Organization, represents the prevailing and highly malignant form of primary brain tumor, exhibiting a 5-year survival rate of 7.2% 2. The prognosis of glioblastoma patients remains poor despite surgical resection and adjuvant chemoradiotherapy 3. Thus, glioblastoma is also regarded as one of the most aggressive and most difficult cancer to treat, and the identification and verification of key molecular in glioblastoma progress are of great significance to develop efficacious targeted therapy 4.

The tetraspanin protein family members (TSPANs) regulate multiple physiological processes, such as cell morphology, adhesion, and motility 5. Most of TSPANs are cell-surface proteins and important mediators of signal transduction across the cell membrane and cell-cell communication 6. Exosomes are cell-cell communicators, and exosomal TSPANs play a vital role in various cellular processes, including the organization and sorting of biomolecules, the selective recruitment of specific molecules, the determination of target selection, the facilitation of cell-specific entry, and the regulation of angiogenesis 7. Transmembrane TSPANs regulate tumor microenvironment (TME) and act a vital role in tumor progression, they can either enhance or inhibit tumor metastasis depend on the multimolecular transmembrane complex 8. Tetraspanin-6 (TSPAN6) is a member of TSPANs and acts as an inhibitor of Ras-driven pancreatic cancer, it interacts with the epidermal growth factor receptor (EGFR) and impedes the activation of RAS induced by EGFR 9. Meanwhile, TSPAN6 acts as a tumor suppressor gene in the progression of colorectal cancer 10. But the function of TSPAN6 on glioblastoma development is still poorly understood. We firstly determined that TSPAN6 acted as an oncogenic gene in glioblastoma progression. Besides, TSPAN6 promoted the angiogenesis of glioblastoma via regulating TME. In addition, TSPAN6 interacted with CDK5 kinase regulatory-subunit associated protein 3 (CDK5RAP3) and regulated STAT3 signaling pathway in glioblastoma. We aimed to provide a key molecular responsible for glioblastoma progression and a potential therapeutics target for glioblastoma treatment.

CDK5RAP3 is originally identified as a binding partner of the CDK5 activator p35, and it has been reported to interact with various proteins, including STAT3, p35, CBP/p300, Rel A, Chk1/2, PAK4, ARF, p38MAPK, UFL1, γ-tubulin, and TIP-1 11, 12. For example, CDK5RAP3 acts as a co-activator of STAT3, and enhances the STAT3-dependent gene expression 13. The function of CDK5RAP3 in cancer progression is controversial depending on tumor type, CDK5RAP3 is overexpressed in hepatocellular cancer and acts as an oncogene in the development of hepatocellular cancer, but it also functions as tumor suppressor in the development of gastric cancer 14, 15.

## Materials and methods

### Materials

The niclosamide (S3030) and mitomycin C (S8146) were obtained from Selleck Chemicals. The antibodies against TSPAN6 (ab236883) and CDK5RAP3 (ab157203) were purchased from Abcam (Waltham, Boston, USA). The antibodies against β-actin (4970), N-cadherin (14215), STAT3 (9139), p-STAT3 (Y-705) (9145), VEGFR2 (9698) were obtained from Cell Signaling Technology (Danvers, Massachusetts, USA). The antibody against E-cadherin (20874-1-AP) was obtained from Proteintech. The antibody against Vimentin (AF0318) was obtained from Beyotime Biotechnology.

### Cell culture

Human glioblastoma cell lines (U87, U251, A172) and human umbilical vein endothelial cells (HUVEC) were obtained from Shanghai cell bank / stem cell bank. U251 and A172 cells were grown in DMEM plus 10% FBS, U87 cells were grown in MEM supplemented with 10% FBS. HUVEC cells were cultured in RPMI-1640 plus 10% FBS.

### SRB assay

After treatment, the cell viability was assessed by SRB staining. Cells were fixed using 10% trichloroacetic acid overnight, washed and treated by SRB solution (0.4%) for 30 min. Then, the dye was removed by 1% acetic acid and dissolved in the Tris-base solution, and cell viability was measured at 515 nm.

### Colony formation assay

After transfection, cells were cultured onto 12-well plates (6000 cells / well). After treatment, the cells were treated with a 1% solution of crystal violet for 30 min and then rinsed with PBS, the images of each well were photographed and colonies were counted.

### Cell migrative and invasive abilities assay by Transwell

The transwell chambers, which had 8-μm pore filter inserts without or with Matrigel, were used to detect the migratory and invasive capabilities of the cells. Cells with serum-free medium were cultured on the upper chamber, and medium plus 20% PBS were cultured onto the bottom chamber. The cells passed through the inserts were fixed / treated for 30 min using crystal violet solution which contained 20% methanol. The migrative and invasive cells were photographed and counted.

### Wound healing assay

The cell migrative ability was also measured using wound healing assay. Cells were cultured onto the plates. After cells were over 90% confluence, scratches were made by sterile pipette tips. The medium was altered and the cells were kept in culture for 24 h. Then, the migrative rate was determined.

### SiRNA and plasmid transfection

The siRNAs were purchased by Genepharma (Shanghai, China), and the sense sequences of siRNA were listed. siTSPAN6-1: 5'-GAGUUGAAGUCAGGGAAUUAUUUCU-3'; siTSPAN6-2: 5'-GCCACAGUCCUUGAAUUGAUGGUAA-3'; siCDK5RAP3: 5'-GCCAGACCAAAGAGAAGAUTT-3'; siSTAT3: 5'-CCACUUUGGUGUUUCAUAATT-3'; negative control siRNA: 5′-UUCUCCGAACGUGUCACGUTT-3′. The TSPAN-6 overexpressing plasmid was constructed and purchased by Aoqian Bio (Hangzhou, China). The cells were introduced with the specified plasmids or siRNA using jetPRIME transfection.

### *In vivo* cell migrative assay

Four weeks old female BALB/c nude mice were purchased from Shanghai SLAC company. Briefly, glioblastoma cells, which had been transfected using indicated shRNA, were injected into nude mice via tail veins (1 × 10^6^ cells per mouse). After 60 days, the mice were euthanized, their lungs were preserved and embedded in paraffin, and haematoxylin and eosin (H&E) staining was used to determine cell migrative ability.

### Western blotting and immunoprecipitation

The procedures of Western blotting and immunoprecipitation were carried out according to previous studies 16.

### LC-ESIMS/MS analysis by Q Exactive HF and Silver Staining

The proteins that were captured through immunoprecipitation were separated using SDS-PAGE and identified through LC-ESIMS/MS analysis conducted by Micrometer Biotech Company in Hangzhou, China. Additionally, protein samples were collected for SDS-PAGE gel electrophoresis after immunoprecipitation, and a silver staining experiment was carried out using the Fast Silver Stain Kit from Beyotime (P0017S).

### mRNA library construction and sequencing

Total RNA was extracted from clinical glioblastoma sample and paracancerous tissue by TRIzol reagent. RNA concentration and purity was determined by NanoDrop (Wilmington, DE, USA). LC-Bio Technology (Hangzhou, China) conducted the construction and sequencing of the mRNA library. The vendor's recommended protocol was followed to conduct RNA sequencing using an Illumina Novaseq™ 6000 in San Diego, CA, USA.

### Immunohistochemistry

The paraffin was removed from glioblastoma and adjacent cancerous tissue using xylene and then the tissues were rehydrated with a series of alcohol solutions. Endogenous peroxidase activity was inhibited using 3% H_2_O_2_. Antigen retrieval was performed by heating the tissue in citrate buffer. The tissue sections were then exposed to the primary antibody at 4°C overnight. A secondary antibody conjugated with horseradish peroxidase was applied at room temperature for 30 minutes, followed by the use of 3,3′-diaminobenzitine (DAB) solution to produce a color reaction.

### The statistics analysis

The data was displayed as mean ± standard deviation. The distinction between groups was examined using one-way ANOVA analysis in GraphPad Prism 9. *** indicates p<0.001, ** indicates p<0.01, and * indicates p<0.05.

## Results

### TSPAN6 overexpression predicts poor outcome of glioma patients

**Figure 1:**
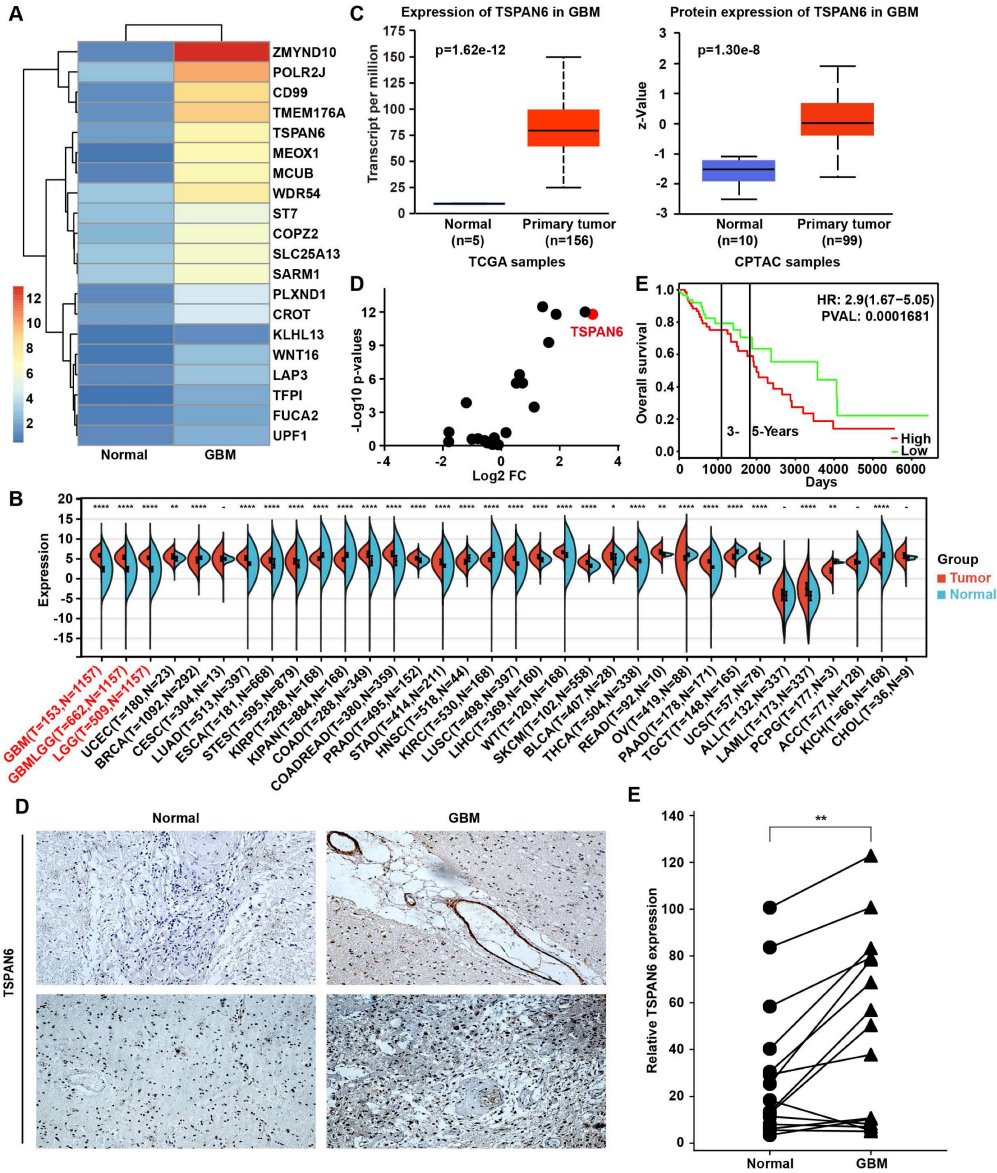
** High level of TSPAN6 is corelated with poor outcomes of glioblastoma patients.** (A) The mRNA expression in glioma tumor tissues was compared to adjacent normal tissues using RNA sequencing (n=1). (B) The result was acquired from Sangerbox 3.0. gene: TSPAN6; datasets: TCGA + GTEx. (C) The result was acquired from UALCAN. Gene: TSPAN6; TCGA dataset: Glioblastoma multiforme; CPTAC dataset: Glioblastoma multiforme. (D) The data were acquired from UALCAN. Gene: TSPAN1-TSPAN33; TCGA dataset: Glioblastoma multiforme; Sample type: Expression cancer stages. (E) The result was obtained from PROGgeneV2 (http://www.progtools.net/gene/). Gene: TSPAN6; Cancer type: Brain; Survival measure: Death; Bifurcate gene expression at median. (F) The immunohistochemistry was performed to detect the expression of TSPAN6 in glioblastoma tissues and adjacent normal tissues.

To explore the carcinogenesis of glioma, this study compared the mRNA expression between glioma tissue and adjacent normal tissue using RNA sequence, and the TOP 20 overexpressed genes in glioma were listed (Figure 1A). UALCAN/GEPIA online tools were applied to further verify whether these genes were statistically overexpressed in glioma compared with normal tissues (Table S1) 17, 18. We identified 12 overexpressed genes in glioblastoma samples compared with normal tissue, including ZMYND10, TFPI, CD99, TMEM176A, TSPAN6, POLR2J, KLHL13, LAP3, COPZ2, CROT, FUCA2, and ST7. TSPAN6 expression was remarkably high in glioma tumor tissues compared to corresponding non-tumor tissues across multiple cancer types using pan-cancer analysis (Figure 1B)^19^. Meanwhile, the mRNA and the protein expressions of TSPAN6 were both overexpressed in glioblastoma compared with normal tissues (Figure 1C)^20^. More importantly, we also determined the expression of TSPANs in glioblastoma and normal tissues, among all the TSPANs, TSPAN6 was mostly overexpressed in glioblastoma comparing to normal tissues (Figure 1D). Furthermore, TSPAN6 overexpression predicted poor outcome of glioma patients (Figure 1E)^21^. Meanwhile, the use of pan-cancer analysis revealed that TSPAN6 expression could serve as a promising prognostic biomarker for glioma patients, as indicated by the COX regression analysis (overall survival: HR = 2.42, p = 7.7e-17; disease-specific survival: HR = 2.55, p = 2.5e-16; progression-free survival: HR = 1.88, p = 8.7e-13; Figure S1-3). Furthermore, the immunohistochemistry results also supported that TSPAN6 was overexpressed in glioblastoma tissues compared with normal tissues (p = 0.0034; Figure 1F). Overall, the above results indicate that TSPAN6 is closely correlated with glioblastoma progress.

### TSPAN6 promotes cell proliferation of glioblastoma cells

**Figure 2:**
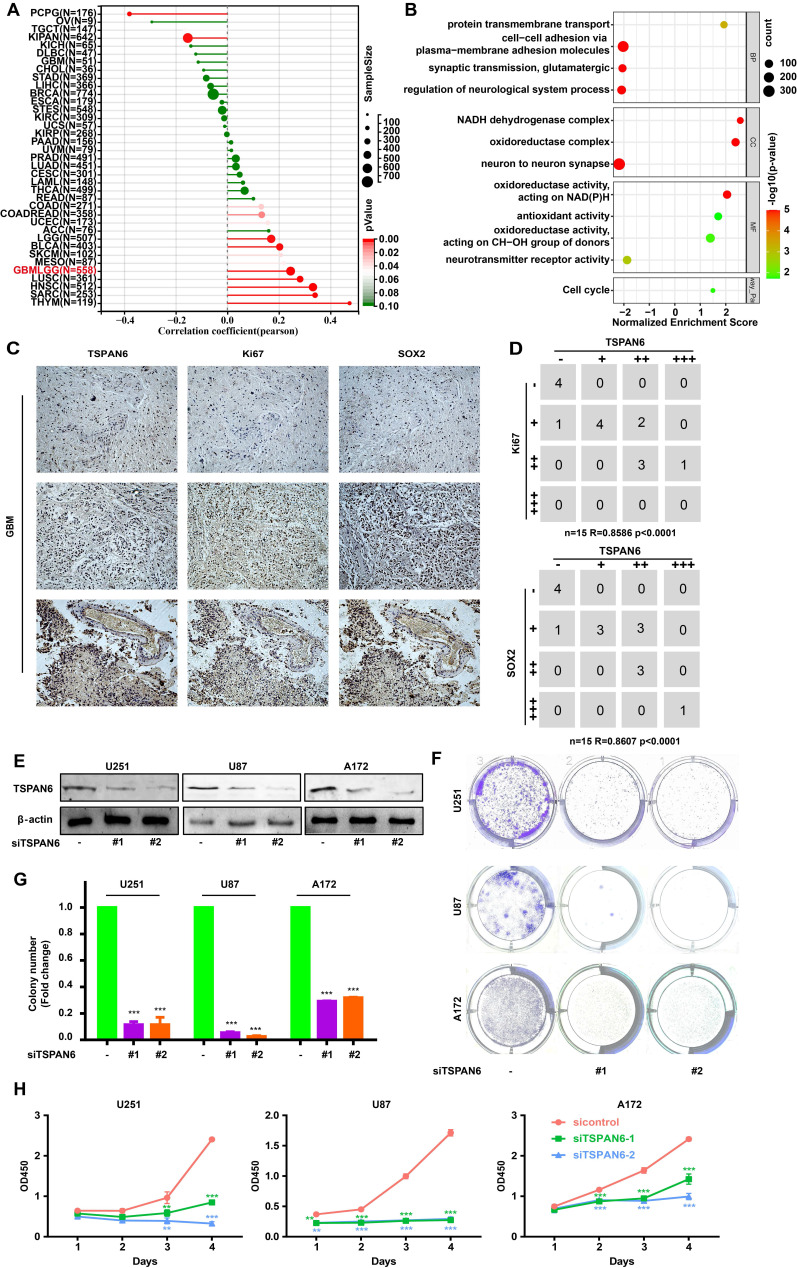
** TSPAN6 promotes cell proliferation of glioblastoma cells.** (A) The result was obtained from Sangerbox 3.0. (B) The result was collected from LinkedOmics. (C) The expression of indicated proteins in glioblastoma were determined by immunohistochemistry. (D) The expression of specific proteins was measured on glioblastoma cells after the 48 h treatment of control siRNA and siTSPAN6. (E-F) Glioblastoma cells were treated with indicated siRNA for 24 h, and colony formation was determined after 14 days. (G) Glioblastoma cells were incubated with indicated siRNA, and the cell proliferation was measured.

The presence of TSPAN6 was positively associated with cancer stem cell-like properties in glioblastoma (Figure 2A). In addition, the presence of TSPAN6 was positively associated with cell cycle of glioblastoma cells (Figure 2B). Meanwhile, the immunohistochemistry results also supported that TSPAN6 could coexpress with stem cell marker Sox2 and division marker Ki67 (r = 0.86, p<0.0001) in glioblastoma tissues (Figure 2C). Furthermore, TSPAN6 suppression significantly restrained the colony formation of glioblastoma cells (Figure 2D-F). Meanwhile, TSPAN6 siRNA significantly restrained cell proliferation of glioblastoma compared with control siRNA (Figure 2G). Thus, TSPAN6 might be involved in the proliferation of glioblastoma cells.

### TSPAN6 reinforces the migration and invasion of glioblastoma cells

**Figure 3:**
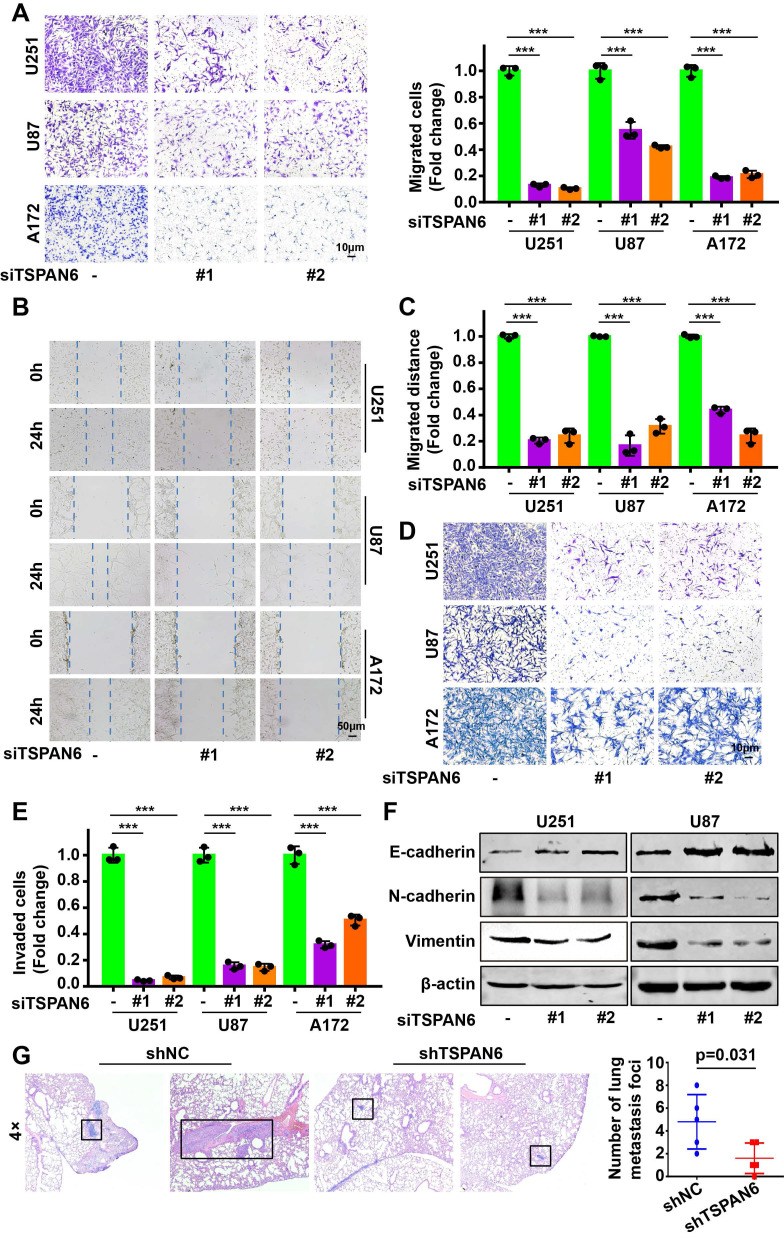
** TSPAN6 silence suppresses the metastatic potential of glioblastoma cells.** (A) Glioblastoma cells were incubated with control siRNA and TSPAN6 siRNA, after 24 h, the cell migration was measured. To inhibit the growth of glioblastoma cells, mitomycin C (10 μg/mL) was introduced. (B-C) The migration of glioblastoma cells was assessed by incubating them with control siRNA and TSPAN6 siRNA for 24 h, followed by a wound healing assay. (D-E) Glioblastoma cells were treated with control siRNA and TSPAN6 siRNA for 24 h, and the cell invasion was determined. (F) The expression of specific proteins was determined in glioblastoma cells after treatment with control siRNA and TSPAN6 siRNA for 48 h. (G) Glioblastoma cell lines stably expressing shRNA targeting TSPAN6 transcripts or negative control shRNA were injected into nude mice via tail vein, and the lung of nude mice was examined for metastatic nodules, which were then stained using H&E. The number of metastatic nodules was then tallied, with a sample size of 5 per group.

**Figure 4:**
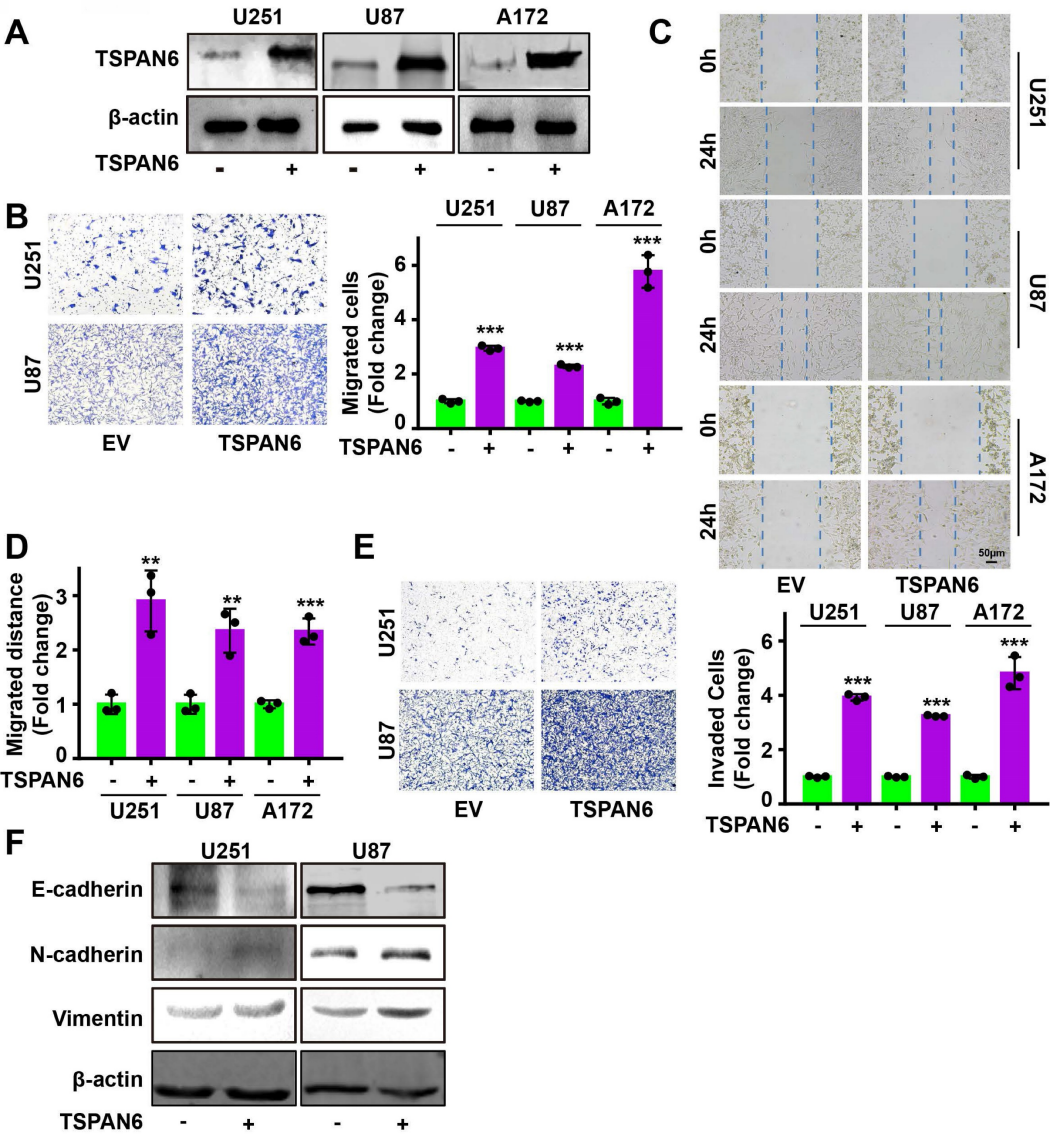
** TSPAN6 reinforces the migration and invasion of glioblastoma cells.** (A) After 48 h transfection, the level of TSPAN6 was determined in glioblastoma cells. (B) After 24 h transfection, the migration of glioblastoma cells was measured. (C-D) After 24 h transfection, the migration of glioblastoma cells was measured. (E) After 24 h transfection, the invasion of glioblastoma cells was determined using Transwell assay. (F) After 48 h transfection, the expression of specific proteins was detected in glioblastoma cells.

Knockdown of TSPAN6 significantly suppressed the migratory ability of glioblastoma cells using transwell assay (Figure 3A-B). Meanwhile, wound healing assay also demonstrated that TSPAN6 silence could restrain the migrative ability of glioblastoma cells (Figure 3C-D). Furthermore, TSPAN6 siRNA statistically restrained the invasive ability of glioblastoma cells compared to control siRNA (Figure 3E). In addition, TSPAN6 knock down significantly suppressed the progression of EMT via enhancing E-cadherin expression and inhibiting N-cadherin/Vimentin expression (Figure 3F). *In vivo* tail vein injection model also confirmed that TSPAN6 knockdown could reduce glioblastoma cells metastasize to lung (Figure 3G). In contrast, TSPAN6 overexpression significantly enhanced the migratory ability of glioblastoma cells (Figure 4A-C, and Figure S4A). In addition, the overexpression of TSPAN6 reinforced the invasion of glioblastoma cells (Figure 4D-E). Furthermore, TSPAN6 elicited the progress of EMT via downregulation of N-cadherin and upregulation of E-cadherin and Vimentin in glioblastoma cells (Figure 4F). Thus, TSPAN6 reinforces the metastatic potential of glioblastoma cells.

### TSPAN6 promotes angiogenesis of glioblastoma

**Figure 5:**
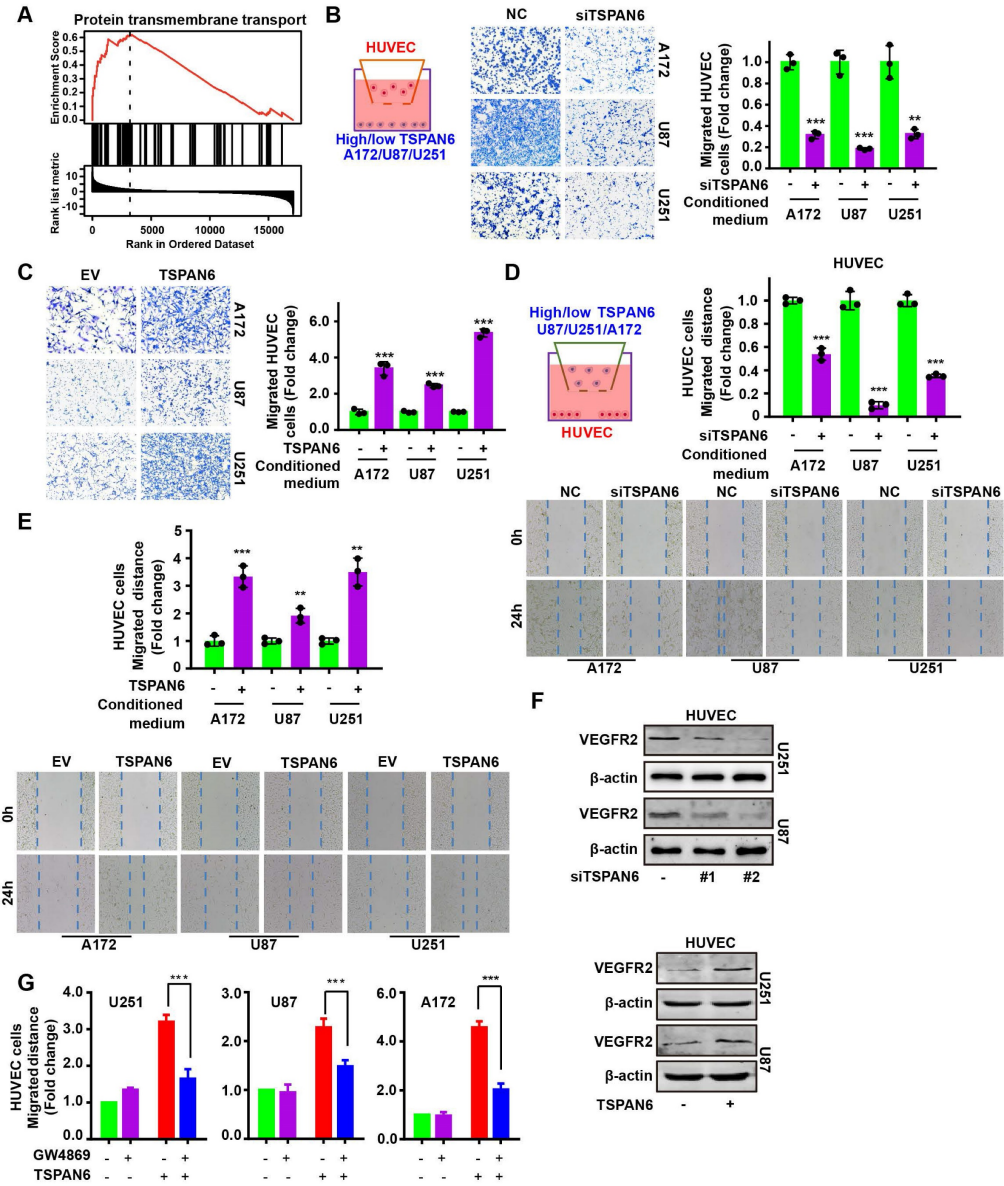
** TSPAN6 promotes angiogenesis of glioblastoma.** (A) The result was collected from LinkedOmics. (B-C) After 48 h transfection and serum-starved overnight, 1 × 10^5^ cancer cells were seeded onto each well. HUVEC cells (1 × 10^4^) were also seeded onto the upper chamber of a Transwell (8.0 µm membrane), and cell migration was assessed after 24 h. (D-E) After 48 h transfection and serum-starved overnight, cancer cells were then seeded onto the upper chamber of a Transwell (0.4 µm membrane). HUVEC cells were also seeded onto each well, and cell migration was assessed using the wound-healing assay. (F) The glioblastoma cells were treated with siRNA or plasmid for 48 h and then placed onto the upper chamber of a Transwell with a polycarbonate membrane that had 0.4 µm pores. HUVEC cells were also seeded onto each well and co-cultured with the glioblastoma cells for 48 h. Western blot analysis was performed to detect the specific protein in the HUVEC cells. (G) The glioblastoma cells were exposed to an empty vector and a plasmid that overexpresses TSPAN6 for 48 h. They were then deprived of serum overnight and placed in the upper chamber of a Transwell (0.4 µm pores). HUVEC cells were seeded in each well at a density of 3 × 10^4^ and treated with either DMSO or GW4869 for 24 h. Cell migration was assessed.

GSEA analysis demonstrated that the expression of TSPAN6 was positively correlated with protein transmembrane transport in glioblastoma (Figure 5A). Thus, we hypothesized that TSPAN6 might be involved in angiogenesis via protein transmembrane transport within TME, and promote tumor growth and metastasis. We constructed a coculture system of vascular endothelial cells and tumor cells *in vitro* to simulate the TME using Transwell (Figure 5B, left panel). The migration of HUVEC cells was detected when those cells were co-cultured with glioblastoma cells treated with negative siRNA and TSPAN6 siRNA. Glioblastoma cells with low level of TSPAN6 statistically reduced the migrative ability of HUVEC cells by regulating TME (Figure 5B, right panel). In contrast, glioblastoma cells with a high level of TSPAN6 enhanced the migrative ability of the vascular endothelial cells (Figure 5C). Meanwhile, glioblastoma cells with TSPAN6 knockdown also decreased the migrative ability of vascular endothelial cells using the wound healing assay, and glioblastoma cells with TSPAN6 overexpression enhanced the migrative ability of vascular endothelial cells (Figure 5D and 5E). Furthermore, TSPAN6-knockdown glioblastoma cells significantly suppressed the level of VEGFR2 in HUVEC cells compared with negative control siRNA transfected glioblastoma cells (Figure 5F, upper panel). In contrast, TSPAN6 overexpressing glioblastoma cells reinforced the expression of VEGFR2 in HUVEC cells compared with empty vector transfected glioblastoma cells (Figure 5F, lower panel). After treated with GW4869 to restrain the exosomes release, glioblastoma cells with TSPAN6 overexpression failed to enhance the migrative ability of vascular endothelial cells (Figure 5G and Figure S5). These data indicate that TSPAN6 overexpression in glioblastoma cells promote angiogenesis via regulating the release of exosomes.

### TSPAN6 interacts with CDK5RAP3 and promotes metastatic potential of glioblastoma cells

**Figure 6:**
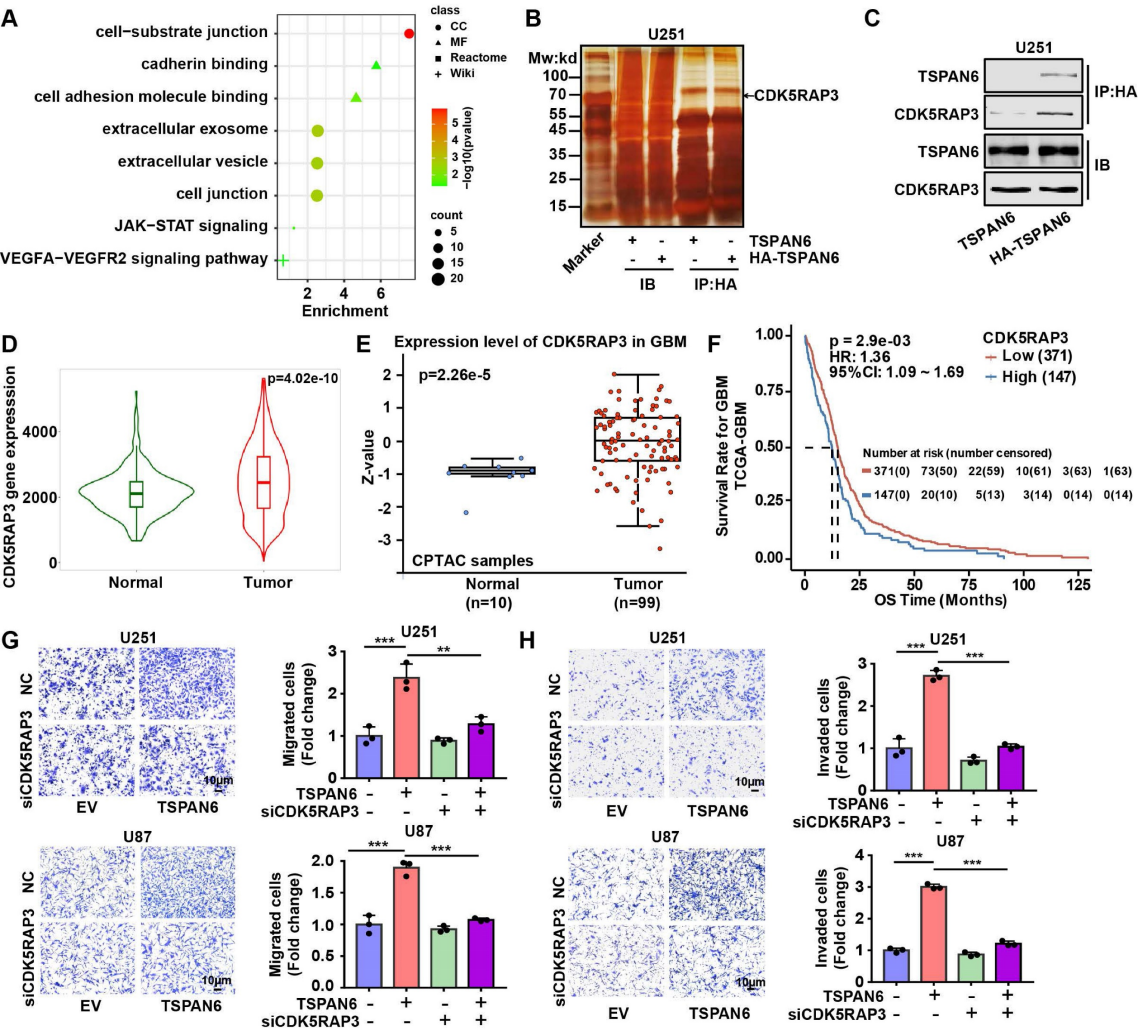
** TSPAN6 interacts with CDK5RAP3 and promotes metastatic potential of glioblastoma cells.** (A) The gene enrichment of TSPAN6-interacting proteins in glioblastoma cells were performed by GO enrichment and String online tools. (B-C) Glioblastoma cells were transfected with TSPAN6 or TSPAN6-HA overexpressing plasmid for 48 h, cell lysate was immunoprecipitated by HA antibody. Subsequently, silver staining (B) and western blot (C) were used to identify the interaction between TSPAN6 and CDK5RAP3. (D) The data was obtained from TNMplot. Gene chip data; Gene: CDK5RAP3; platform: use non-paired tumor and normal tissues; Tissue: CNS. (E) The result was obtained from UALCAN. CPTAC; Gene: CDK5RAP3; CPTAC dataset: glioblastoma. (F) The data was obtained from PanCanSurvPlot (https://smuonco.shinyapps.io/PanCanSurvPlot/). Gene: CDK5RAP3; Cancer: glioblastoma; Dataset: TCGA-glioblastoma; Platform: AffyU133a; Survival: OS. (G-H) The migrative and invasive abilities of glioblastoma cells with a high level of TSPAN6 were assessed after transfection with control siRNA and CDK5RAP3 siRNA for 24 h.

To investigate the mechanism of TSPAN6 in the progress of glioblastoma, we identified 89 TSPAN6-interacting proteins in glioblastoma cells using LC-MS/MS (Table S2). The gene enrichment of TSPAN6-interacting proteins demonstrated that TSPAN6 was correlated with cell adhesion, cell junction, extracellular exosomes, VEGFA-VEGFR2 and JAK-STAT signaling pathway (Figure 6A and Table S3) 22, 23. Thus, TSPAN6 might promote metastatic potential and angiogenesis of glioblastoma via exosomes. Because CDK5RAP3 was identified as a potential TSPAN6-interacting protein in glioblastoma cells using LC-MS/MS, and TSPAN6 might be involved in JAK-STAT signaling pathway via gene enrichment analysis of TSPAN6-interacting proteins. Meanwhile, it was also reported that CDK5RAP3 was a co-factor for the oncogenic transcription factor STAT3 and mediated tumorigenic phenotypes such as proliferation and migration 13. Thus, we assumed that TSPAN6 might interact with CDK5RAP3 and enhance the progress of glioblastoma via STAT3.

As shown in Figure 6B, the physical interaction between TSPAN6 and CDK5RAP3 could be confirmed using immunoprecipitation followed by silver staining. Furthermore, we also used immunoprecipitation to show evidence of direct molecular interaction between TSPAN6 and CDK5RAP3 in U251 cells (Figure 6C). In addition, the mRNA and protein levels of CDK5RAP3 were overexpressed in glioblastoma clinical samples compared with normal tissues (Figure 6D and 6E) 24. The overexpression of CDK5RAP3 was corelated with poor overall survival of glioblastoma patients (Figure 6F) 25. Furthermore, CDK5RAP3 knockdown successfully reversed the enhanced migrative and invasive abilities induced by TSPAN6 overexpression in glioblastoma cells (Figure 6G and 6H, Figure S4B). Thus, TSPAN6 may interact with CDK5RAP3 and promote metastatic potential of glioblastoma cells.

### TSPAN6 interacts with CDK5RAP3 and activates STAT3 signaling pathway

**Figure 7:**
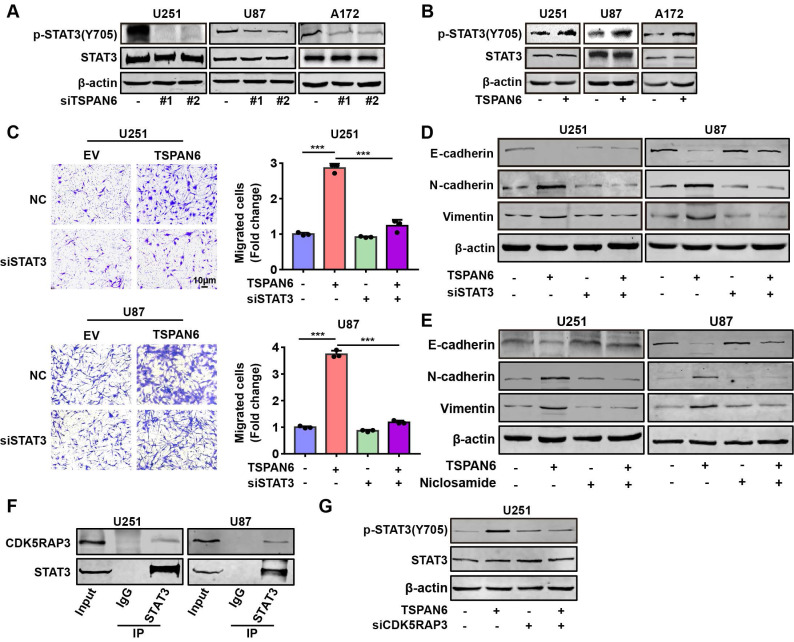
** TSPAN6 interacts with CDK5RAP3 and activates STAT3 signaling pathway.** (A-B) After 48 h transfection, the expression of specific proteins in glioblastoma cells was examined by western blot. (C-D) After 24 transfection with siRNA, the migrative abilities of glioblastoma cells and the expression of EMT related proteins were assessed. (E) Glioblastoma cells with high level of TSPAN6 were treated with DMSO or 5 μM niclosamide, and western blot was used to measure the protein expression. (F) Immunoprecipitation assay revealed the physical interaction of CDK5RAP3 with STAT3 in glioblastoma cells. (G) After 24 transfection with siRNA, the level of specific proteins was measured in glioblastoma cells that had a high level of TSPAN6.

Next, we hypothesized TSPAN6 might activate STAT3 signaling pathway via interacting with CDK5RAP3. TSPAN6 knockdown statistically inhibited the activation of STAT3 in glioblastoma cells (Figure 7A). In contrast, TSPAN6 overexpression promoted the activation of STAT3 via enhancing the phosphorylating STAT3 in glioblastoma cells (Figure 7B). Furthermore, STAT3 silence significantly alleviated the enhanced cell migration capacity induced by TSPAN6 overexpression (Figure 7C, Figure S4C). Meanwhile, STAT3 knockdown also reversed TSPAN6 activated EMT progress of glioblastoma cells (Figure 7D). In addition, STAT3 inhibitor niclosamide successfully reversed TSPAN6 activated EMT progress of glioblastoma cells (Figure 7E). As we expected, the direct interaction between CDK5RAP3 and STAT3 could also be verified in glioblastoma cells using immunoprecipitation (Figure 7F). Moreover, CDK5RAP3 knockdown could efficiently reverse TSPAN6-induced STAT3 activation in glioblastoma cells (Figure 7G). These data suggest that TSPAN6 may activate STAT3 signaling pathway via CDK5RAP3 in glioblastoma cells.

### TSPAN6 promotes angiogenesis of glioblastoma via activating STAT3

**Figure 8:**
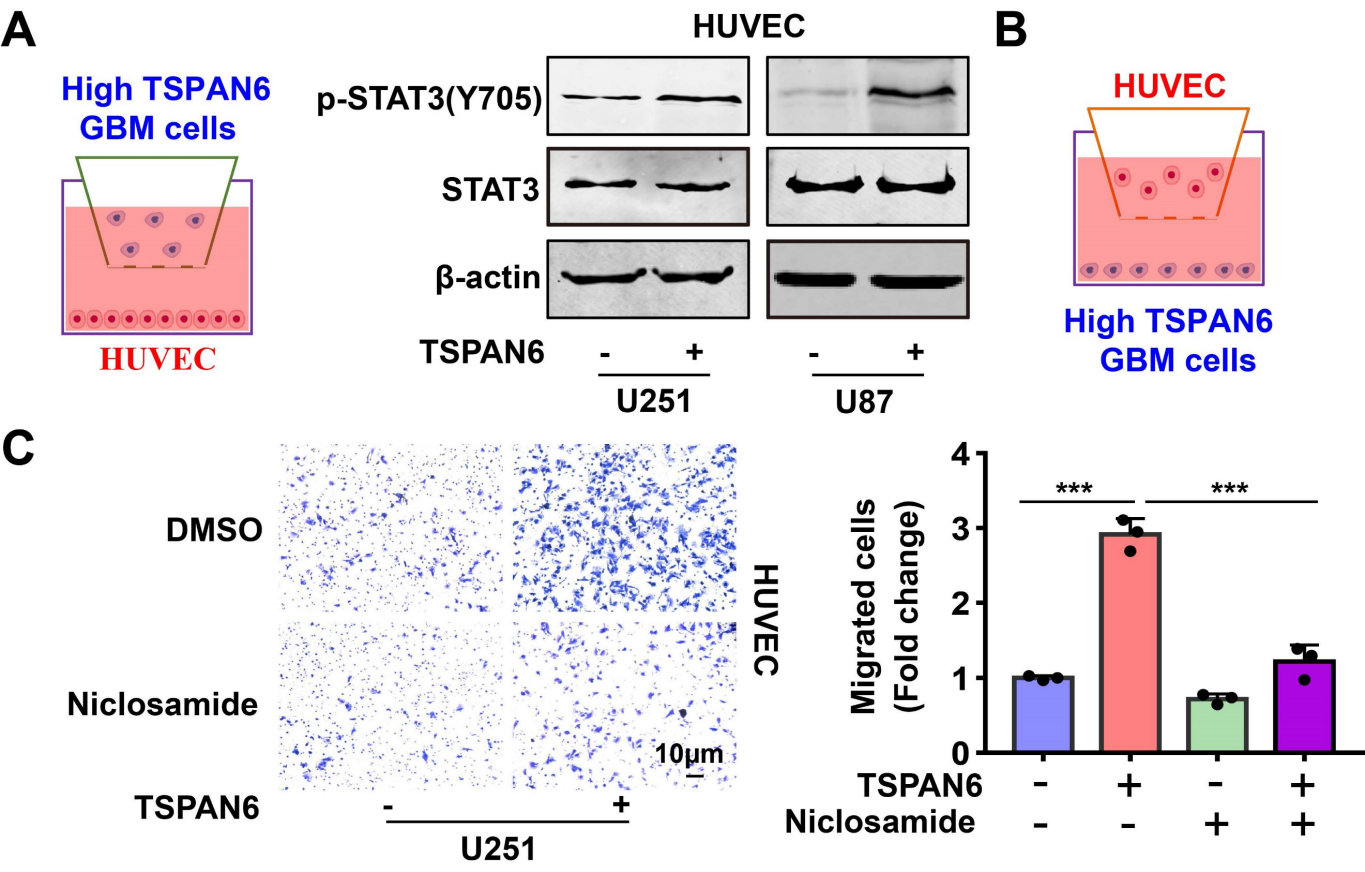
** TSPAN6 promotes angiogenesis of glioblastoma via activating STAT3.** (A) The glioblastoma cells were exposed to either an empty vector or a plasmid that overexpresses TSPAN6 for 48 h. Then, they were placed in the upper chamber of a Transwell with a polycarbonate membrane that has 0.4 µm pores. HUVEC cells were also seeded in each well and co-cultured with the glioblastoma cells for 48 h. Western blot analysis was performed to identify the specific protein in the HUVEC cells. (B-C) The glioblastoma cells were exposed to either an empty vector or a plasmid that overexpresses TSPAN6 for 48 h. Then, they were placed in each well at a concentration of 1 × 10^5^ in 500 μL of culture medium without serum. HUVEC cells (1 × 10^4^) were placed in the upper chamber of a Transwell with a polycarbonate membrane with 8.0 µm pores. These HUVEC cells were treated with 1 μM of niclosamide, and their ability to migrate was assessed after 24 h.

To explore the mechanism of TSPAN6 in promoting angiogenesis in TME, we hypothesized that glioblastoma cells overexpressing TSPAN6 might induce STAT3 activation of vascular endothelial cells. Indeed, glioblastoma cells with high level of TSPAN6 statistically enhanced STAT3 activation of HUVEC cells (Figure 8A). Meanwhile, STAT3 inhibitor niclosamide suppressed the enhanced migrative ability induced by glioblastoma cells overexpressing TSPAN6 in HUVEC cells (Figure 8B and 8C). Thus, glioblastoma cells overexpressing TSPAN6 may induce STAT3 activation of vascular endothelial cells and promote angiogenesis in TME of glioblastoma.

## Discussion

In most cases of glioblastoma, patients usually have no clinical symptoms, and glioblastoma patients are usually diagnosed in the middle or late stages of the disease which makes radical surgery challenging 26. Furthermore, glioblastoma treatment is hampered by diffuse tumor cell invasion and metastasis, triggering cancer cells to migrate away from the tumor core area into the brain parenchyma and escape from surgical resection 27. Tumors therefore reoccur locally in up to 90% of glioblastoma cases despite aggressive multimodal treatment approaches 28. Thus, innovative drugs are urgently needed to treat advanced metastatic glioblastoma. Despite several drug-target engagement, attempts to directly suppress oncogenic pathways in glioblastoma have failed to translate to meaningful breakthroughs in clinical treatment 29. The treatment glioblastoma has remained almost unchanged for more than 20 years, which may partially due to the heterogeneity of glioblastoma 30.

The TME of glioblastoma provides a dynamic array of signals that drive cancer progression and thus is being regarded as a therapeutic target to disrupt angiogenesis or immunosuppression 31. TSPANs are consist of 24 family genes and involved in cancer progress, for example, TSPAN4 is regarded as a potential prognostic and immune target in glioblastoma, and it also serves as a potential biomarker and the crosstalk between atherosclerosis and tumor progression 32. Meanwhile, TSPANs are also important actors of cell communication in TME, and they are abundantly enriched in exosomes as transmembrane proteins 33, 34. For instance, TSPAN6 is increased in AD brains and is a pivotal protein determining exosome release 35. TSPANs show conflicting results on expression in different tumor types, it has been reported that TSPAN6 acts as a tumor suppressor gene in the development of colorectal and pancreatic cancer 9, 10. So far, the function of TSPAN6 in glioblastoma development has not been elucidated. We established RNA sequence to compare the mRNA expression between clinical glioma tumor tissue and adjacent normal tissue, TSPAN6 was the only gene among TSPANs which was listed on TOP 20 upregulated genes in glioma. This study also firstly demonstrated that TSPAN6 was remarkably high in glioma tumor tissues compared to corresponding non-tumor tissues using pan-cancer analysis based on TCGA database. Meanwhile, TSPAN6 expression was a potential prognostic biomarker for glioma patients. Furthermore, TSPAN6 reinforced the cell proliferative ability and metastatic potential of glioblastoma cells, indicating that TSPAN6 might be a potential therapeutics target for glioblastoma. More interestingly, TSPAN6 overexpression in glioblastoma cells promoted angiogenesis via enhancing the migrative ability of vascular endothelial cells in TME. Thus, TSPAN6 is also regarded as a therapeutic target to regulate TME of glioblastoma, and the significance of TSPAN6 in cancer progress may provide clues for the diagnosis and treatment of glioblastoma.

CDK5RAP3 is originally identified as a binding protein of Cdk5 activator p35, and it is involved in a wide variety of signaling pathways, including NF-κB, ARF/p53, Wnt, STAT3, DNA damage response and UFMylation 11. However, CDK5RAP3 plays a multifaceted role in tumorigenesis which may depending on cancer type, for example, CDK5RAP3 acts as a tumor suppressor gene in gastric cancer, while it acts as an oncogene in hepatocellular carcinoma 15, 36. However, the role of CDK5RAP3 in the progress of glioblastoma is not well established. In this study, we firstly identified the CDK5RAP3 was a TSPAN6-interacting proteins in glioblastoma cells. Meanwhile, CDK5RAP3 was overexpressed in glioblastoma compared with normal tissues, and CDK5RAP3 overexpression predicted poor outcomes of glioblastoma patients. Furthermore, TSPAN6 interacted with CDK5RAP3 and promoted the progress of glioblastoma. However, the molecular mechanism of TSPAN6 in interacting with CDK5RAP3 might need further investigation.

STAT3 is a critical mediator of tumorigenesis and tumor progression of glioblastoma, its activation induces cell proliferation, anti-apoptosis, glioma stem cell maintenance, tumor invasion, angiogenesis, and immune evasion 37. Meanwhile, CDK5RAP3 is identified as a co-factor for STAT3 and mediated tumorigenic phenotypes including clonogenesis and migration 13. Thus, we assumed that TSPAN6 might regulated STAT3 signaling pathway via interacting with CDK5RAP3. This study demonstrated that TSPAN6 enhanced the phosphorylation of STAT3 in glioblastoma cells, CDK5RAP3 knockdown reversed TSPAN6-activated STAT3, indicating that TSPAN6 activated STAT3 via CDK5RAP3 in glioblastoma cells. STAT3 signaling pathway regulates the biogenesis of tumor-derived exosomes contributing to the progress of cancer 38. Furthermore, STAT3 knockdown blocks the angiogenesis via regulating TME 39. In this study, we hypothesized that TSPAN6 might promote the biogenesis of exosomes via STAT3 pathway in glioblastoma cells, and subsequently regulate angiogenesis in TME by STAT3. Indeed, exosome secretion inhibitor reversed the migrative abilities of vascular endothelial cells enhanced by TSPAN6 overexpressing glioblastoma cells. In addition, TSPAN6 induced STAT3 activation of vascular endothelial cells and promoted angiogenesis in TME of glioblastoma. However, the mechanism of TSPAN6 in regulating TME and angiogenesis might need further investigations. Meanwhile, TSPAN6 and STAT3 were both mostly overexpressed in endothelial cells of glioblastoma, and the colocalization of TSPAN6 and STAT3 was verified in endothelial cells by single cell sequencing. STAT3 inhibition suppressed the enhanced migrative ability induced by glioblastoma cells overexpressing TSPAN6 in HUVEC cells. Thus, TSPAN6 promotes cancer progress by STAT3 signaling pathway in glioblastoma cells, and TSPAN6 also regulates angiogenesis via exosome through STAT3 pathway in TME.

Collectively, this work firstly declared that TSPAN6 was overexpressed in glioblastoma and TSPAN6 overexpression predicted poor outcome of glioblastoma patients. Furthermore, TSPAN6 promoted the malignant progression of glioblastoma by interacting with CDK5RAP3 and regulating STAT3. Meanwhile, TSPAN6 enhanced angiogenesis via regulating TME and STAT3 signaling pathway. These data suggests that TSPAN6 may be a potential therapeutic target and prognosis biomarker for glioblastoma treatment.

## Supplementary Material

Supplementary figures and tables.

## Abbreviations

TME: tumor microenvironment
TSPANs: tetraspanin protein family members
TSPAN6: Tetraspanin-6
CDK5RAP3: CDK5 kinase regulatory-subunit associated protein 3
H&E: haematoxylin and eosin

## References

[1] Xu SM, Xiao HY, Hu ZX, Zhong XF, Zeng YJ, Wu YX (2023). GRN is a prognostic biomarker and correlated with immune infiltration in glioma: A study based on TCGA data. Front Oncol.

[2] Wu W, Klockow JL, Zhang M, Lafortune F, Chang E, Jin L (2021). Glioblastoma multiforme (GBM): An overview of current therapies and mechanisms of resistance. Pharmacol Res.

[3] Zhang H, Zhang N, Wu W, Wang Z, Dai Z, Liang X (2022). Pericyte mediates the infiltration, migration, and polarization of macrophages by CD163/MCAM axis in glioblastoma. iScience.

[4] Liu R, Dai W, Wu A, Li Y (2021). CircCDC45 promotes the malignant progression of glioblastoma by modulating the miR-485-5p/CSF-1 axis. BMC Cancer.

[5] Umeda R, Satouh Y, Takemoto M, Nakada-Nakura Y, Liu K, Yokoyama T (2020). Structural insights into tetraspanin CD9 function. Nat Commun.

[6] Zou F, Wang X, Han X, Rothschild G, Zheng SG, Basu U (2018). Expression and Function of Tetraspanins and Their Interacting Partners in B Cells. Front Immunol.

[7] Malla RR, Pandrangi S, Kumari S, Gavara MM, Badana AK (2018). Exosomal tetraspanins as regulators of cancer progression and metastasis and novel diagnostic markers. Asia Pac J Clin Oncol.

[8] Richardson MM, Jennings LK, Zhang XA (2011). Tetraspanins and tumor progression. Clin Exp Metastasis.

[9] Humbert PO, Pryjda TZ, Pranjic B, Farrell A, Fujikura K, de Matos Simoes R (2022). TSPAN6 is a suppressor of Ras-driven cancer. Oncogene.

[10] Andrijes R, Hejmadi RK, Pugh M, Rajesh S, Novitskaya V, Ibrahim M (2021). Tetraspanin 6 is a regulator of carcinogenesis in colorectal cancer. Proc Natl Acad Sci U S A.

[11] Quintero M, Liu S, Xia Y, Huang Y, Zou Y, Li G (2021). Cdk5rap3 is essential for intestinal Paneth cell development and maintenance. Cell Death Dis.

[12] Chen QY, Liu LC, Wang JB, Xie JW, Lin JX, Lu J (2019). CDK5RAP3 Inhibits the Translocation of MCM6 to Influence the Prognosis in Gastric Cancer. J Cancer.

[13] Egusquiaguirre SP, Liu S, Tosic I, Jiang K, Walker SR, Nicolais M (2020). CDK5RAP3 is a co-factor for the oncogenic transcription factor STAT3. Neoplasia.

[14] Lin JX, Xie XS, Weng XF, Zheng CH, Xie JW, Wang JB (2018). Low expression of CDK5RAP3 and DDRGK1 indicates a poor prognosis in patients with gastric cancer. World J Gastroenterol.

[15] Lin JX, Yoon C, Li P, Ryeom SW, Cho SJ, Zheng CH (2020). CDK5RAP3 as tumour suppressor negatively regulates self-renewal and invasion and is regulated by ERK1/2 signalling in human gastric cancer. Br J Cancer.

[16] Li YL, Zhang MM, Wu LW, Liu YH, Zhang ZY, Zeng LH (2022). DYRK1A reinforces epithelial-mesenchymal transition and metastasis of hepatocellular carcinoma via cooperatively activating STAT3 and SMAD. J Biomed Sci.

[17] Chandrashekar DS, Karthikeyan SK, Korla PK, Patel H, Shovon AR, Athar M (2022). UALCAN: An update to the integrated cancer data analysis platform. Neoplasia.

[18] Tang Z, Li C, Kang B, Gao G, Li C, Zhang Z (2017). GEPIA: a web server for cancer and normal gene expression profiling and interactive analyses. Nucleic Acids Res.

[19] Shen W, Song Z, Zhong X, Huang M, Shen D, Gao P (2022). Sangerbox: A comprehensive, interaction-friendly clinical bioinformatics analysis platform. iMeta.

[20] Zhang Y, Chen F, Chandrashekar DS, Varambally S, Creighton CJ (2022). Proteogenomic characterization of 2002 human cancers reveals pan-cancer molecular subtypes and associated pathways. Nat Commun.

[21] Goswami CP, Nakshatri H (2014). PROGgeneV2: enhancements on the existing database. BMC Cancer.

[22] Szklarczyk D, Kirsch R, Koutrouli M, Nastou K, Mehryary F, Hachilif R (2023). The STRING database in 2023: protein-protein association networks and functional enrichment analyses for any sequenced genome of interest. Nucleic Acids Res.

[23] Ashburner M, Ball CA, Blake JA, Botstein D, Butler H, Cherry JM (2000). Gene ontology: tool for the unification of biology. The Gene Ontology Consortium. Nat Genet.

[25] Lin A, Yang H, Shi Y, Cheng Q, Liu Z, Zhang J (2022). PanCanSurvPlot: A Large-scale Pan-cancer Survival Analysis Web Application. bioRxiv. 2022.

[26] Yan X, Feng L, Xu Z, Chen W, Yan H, Wu P Histone acetylation gene-based biomarkers as novel markers of the immune microenvironment in glioblastoma. J Gene Med. 2023: e3511.

[27] Li J, Wang X, Chen L, Zhang J, Zhang Y, Ren X (2022). TMEM158 promotes the proliferation and migration of glioma cells via STAT3 signaling in glioblastomas. Cancer Gene Ther.

[28] Waldherr L, Seitanidou M, Jakesova M, Handl V, Honeder S, Nowakowska M (2021). Targeted Chemotherapy of Glioblastoma Spheroids with an Iontronic Pump. Adv Mater Technol.

[29] Muir M, Gopakumar S, Traylor J, Lee S, Rao G (2020). Glioblastoma multiforme: novel therapeutic targets. Expert Opin Ther Targets.

[30] Bausart M, Preat V, Malfanti A (2022). Immunotherapy for glioblastoma: the promise of combination strategies. J Exp Clin Cancer Res.

[31] Wolf KJ, Chen J, Coombes J, Aghi MK, Kumar S (2019). Dissecting and rebuilding the glioblastoma microenvironment with engineered materials. Nat Rev Mater.

[32] Zheng Y, Lang Y, Qi B, Wang Y, Gao W, Li T (2022). TSPAN4 is a prognostic and immune target in Glioblastoma multiforme. Front Mol Biosci.

[33] Huang R, Sun H, Lin R, Zhang J, Yin H, Xian S (2022). The role of tetraspanins pan-cancer. iScience.

[34] Rana S, Yue S, Stadel D, Zoller M (2012). Toward tailored exosomes: the exosomal tetraspanin web contributes to target cell selection. Int J Biochem Cell Biol.

[35] Guix FX, Sannerud R, Berditchevski F, Arranz AM, Horre K, Snellinx A (2017). Tetraspanin 6: a pivotal protein of the multiple vesicular body determining exosome release and lysosomal degradation of amyloid precursor protein fragments. Mol Neurodegener.

[36] Mak GW, Chan MM, Leong VY, Lee JM, Yau TO, Ng IO (2011). Overexpression of a novel activator of PAK4, the CDK5 kinase-associated protein CDK5RAP3, promotes hepatocellular carcinoma metastasis. Cancer Res.

[37] Chang N, Ahn SH, Kong DS, Lee HW, Nam DH (2017). The role of STAT3 in glioblastoma progression through dual influences on tumor cells and the immune microenvironment. Mol Cell Endocrinol.

[38] Fan M, Sun W, Gu X, Lu S, Shen Q, Liu X (2022). The critical role of STAT3 in biogenesis of tumor-derived exosomes with potency of inducing cancer cachexia in vitro and in vivo. Oncogene.

[39] Liu Y, Luo F, Wang B, Li H, Xu Y, Liu X (2016). STAT3-regulated exosomal miR-21 promotes angiogenesis and is involved in neoplastic processes of transformed human bronchial epithelial cells. Cancer Lett.

