# Supplementary material for: TSPAN6 reinforces the malignant progression of glioblastoma via interacting with CDK5RAP3 and regulating STAT3 signaling pathway
Source: Int J Biol Sci. 2024 Apr 15;20(7):2440–53. doi: 10.7150/ijbs.85984 (PMC11077372; doi:10.7150/ijbs.85984)
Supplement: Supplementary file 1 — Supplementary figures and tables. [file ijbsv20p2440s1.zip › supplementary legends.pdf]

Table S1: The TOP 20 overexpressed genes in glioblastoma compared with normal tissues.

Table S2: The proteins interacted with TSPAN6 identified by LC-MS/MS.

Table S3: The GO enrichment of TSPAN6 interacting proteins in glioblastoma cells.

Figure S1: The expression of TSPAN6 is a potential prognostic biomarker for overall survival of glioma patients. The data was collected from Sangerbox 3.0. Gene: TSPAN6; Survival data: overall survival.

Figure S2: The expression of TSPAN6 is a potential prognostic biomarker for disease-specific survival of glioma patients. The data was collected from Sangerbox 3.0. Gene: TSPAN6; Survival data: disease-specific survival.

Figure S3: The expression of TSPAN6 is a potential prognostic biomarker for progression-free survival of glioma patients. The data was collected from Sangerbox 3.0. Gene: TSPAN6; Survival data: progression-free interval.

Figure S4: The efficacy of gene knockdown and overexpression in glioblastoma cells. (A) Glioblastoma cells were transfected with empty vector and TSPAN6 overexpressing plasmid for 48 h, and the mRNA level of TSPAN6 was detected. (B) Glioblastoma cells were transfected with control siRNA and siCDK5RAP3 for 48 h, and the protein level of CDK5RAP3 was detected. (C) Glioblastoma cells were transfected with control siRNA and siSTAT3 for 48 h, and the protein level of STAT3 was detected.

Figure S5: TSPAN6 promotes angiogenesis of glioblastoma. Glioblastoma cells were incubated with empty vector and TSPAN6 overexpressing plasmid for 48 h, serum-starved overnight, and seeded at a density of 1×10^5/well onto the upper chamber of the Transwell with 0.4 μm pore polycarbonate membrane. HUVEC cells were seeded onto each well at a density of 3×10^4 and treated with DMSO or GW4869 for 24 h, and the migration ability of HUVEC cells were determined by using the wound-healing assay.
