# Supplementary figures and images for: TSPAN6 reinforces the malignant progression of glioblastoma via interacting with CDK5RAP3 and regulating STAT3 signaling pathway
Source: Int J Biol Sci. 2024 Apr 15;20(7):2440–53. doi: 10.7150/ijbs.85984 (PMC11077372; doi:10.7150/ijbs.85984)

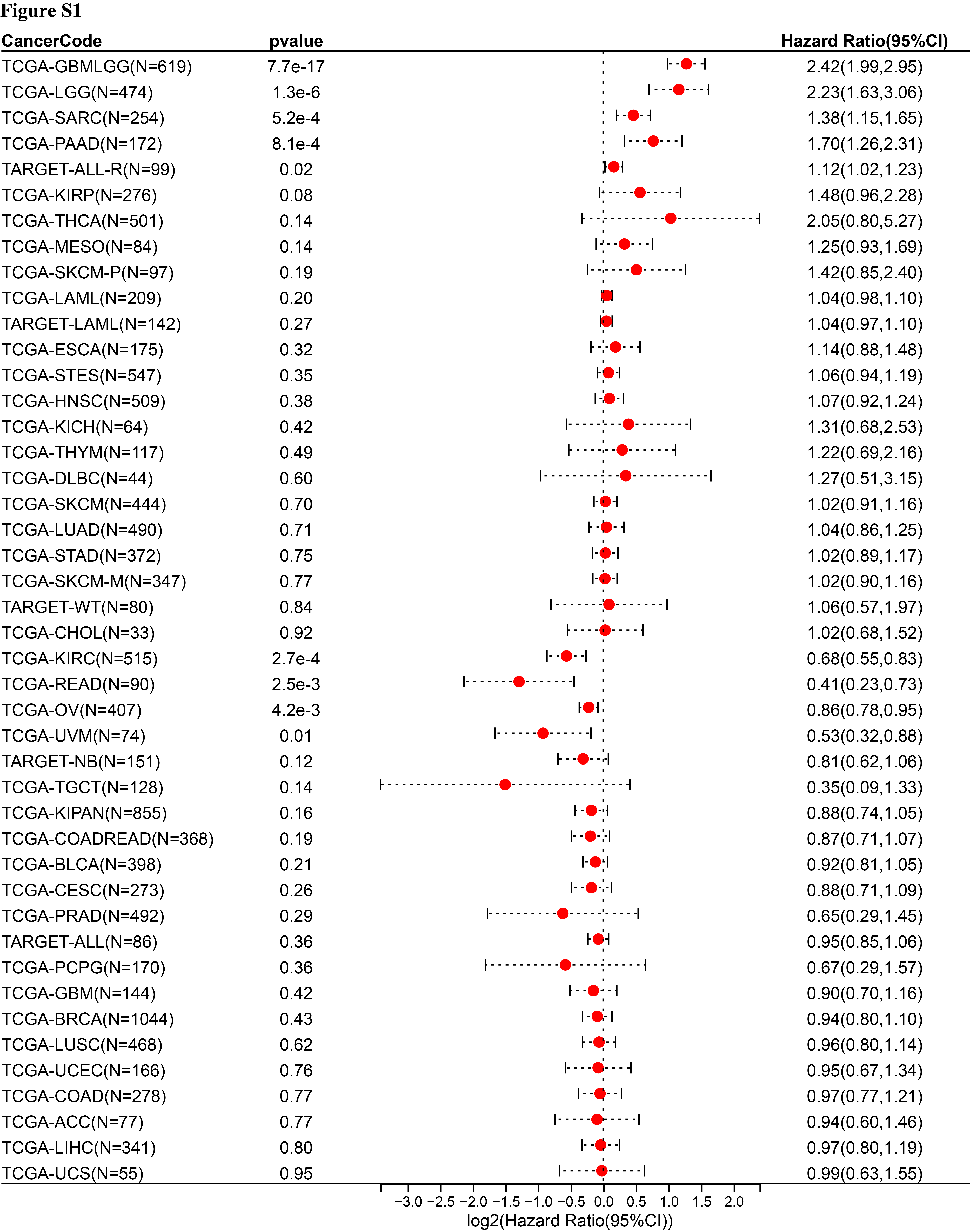

Supplement: Supplementary file 1 — Supplementary figures and tables. [file ijbsv20p2440s1.zip › Supplementary figure 1-OS.jpg]

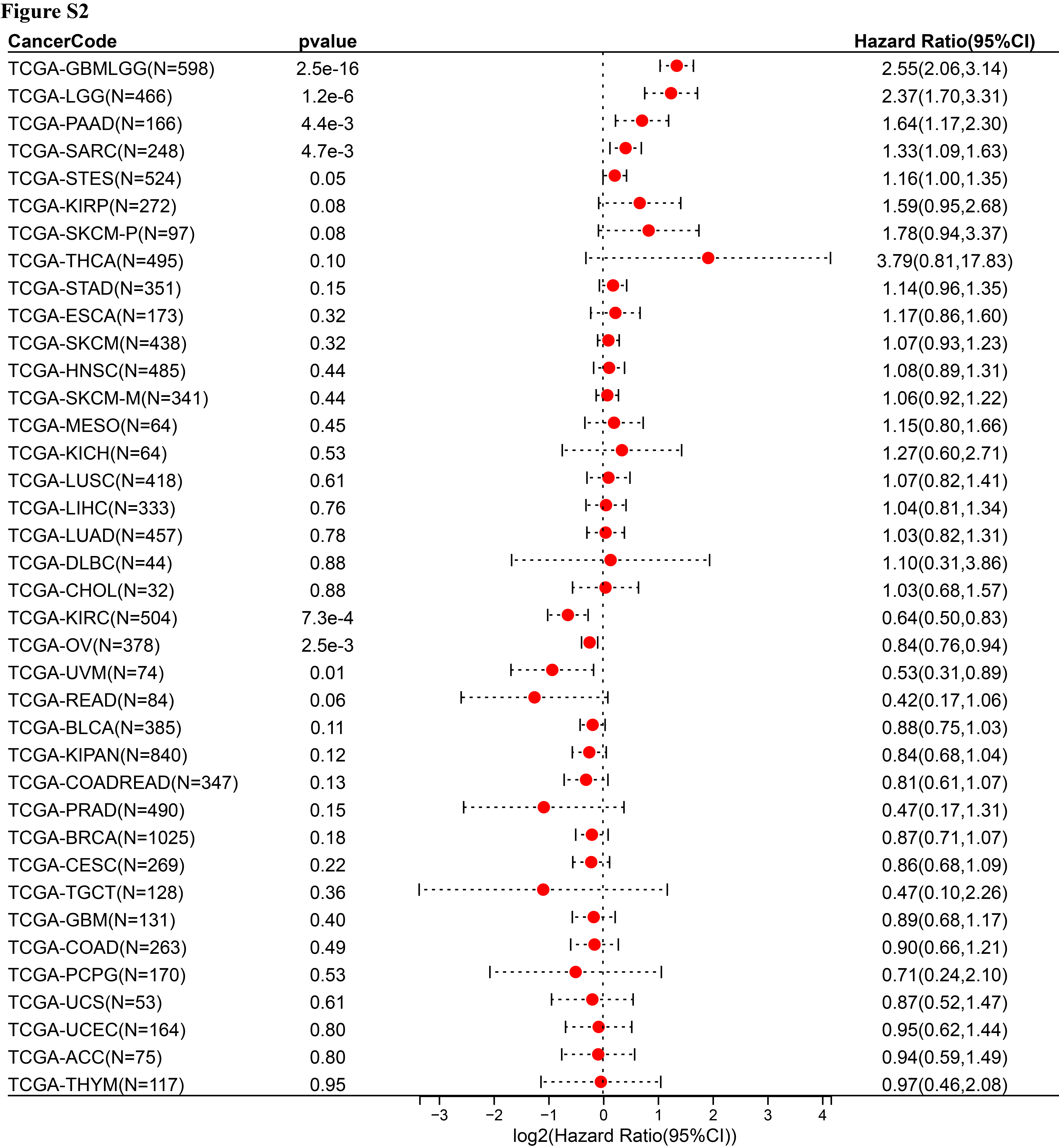

Supplement: Supplementary file 1 — Supplementary figures and tables. [file ijbsv20p2440s1.zip › Supplementary figure 2-DSS.jpg]

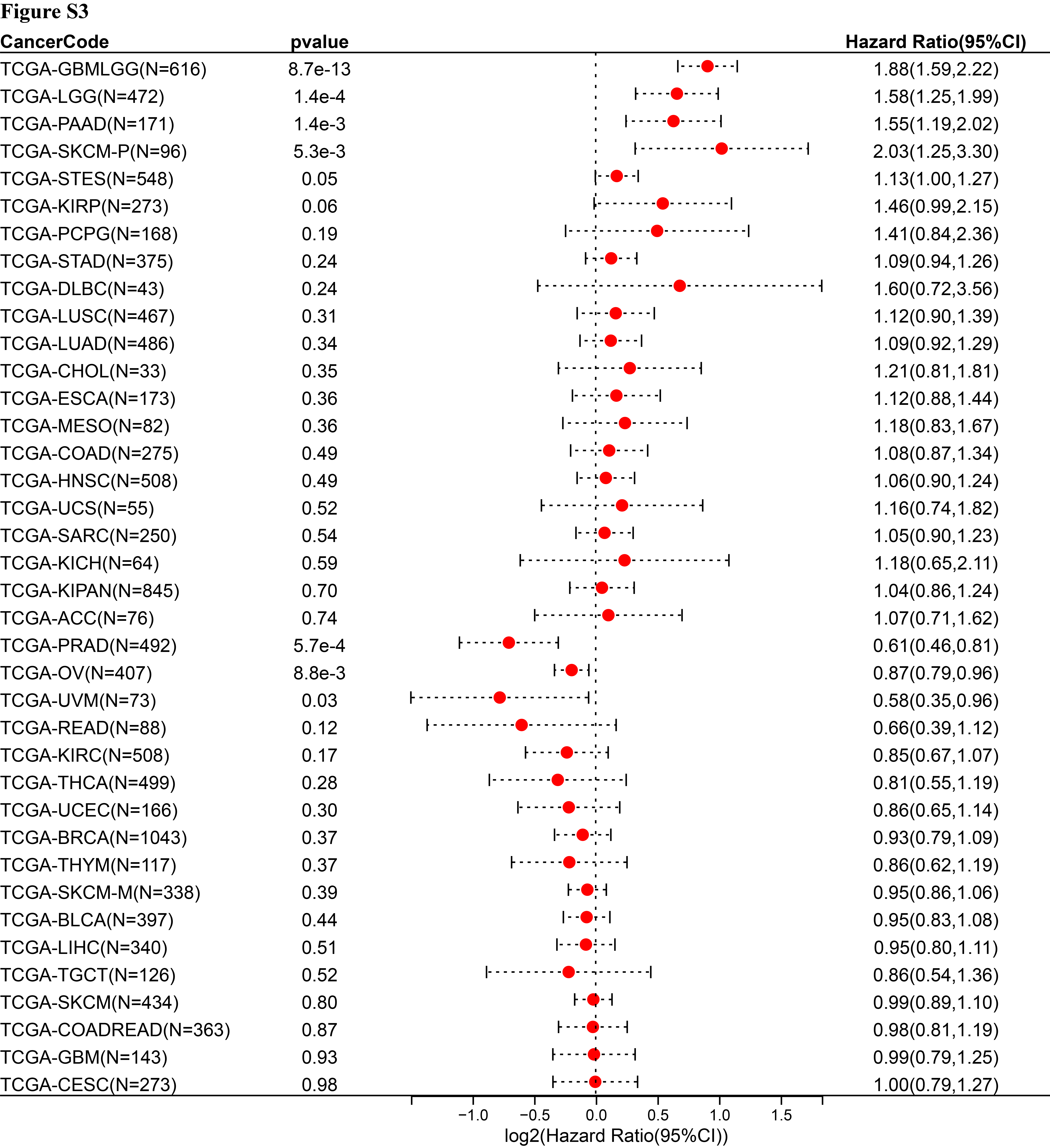

Supplement: Supplementary file 1 — Supplementary figures and tables. [file ijbsv20p2440s1.zip › Supplementary figure 3-PFS.jpg]

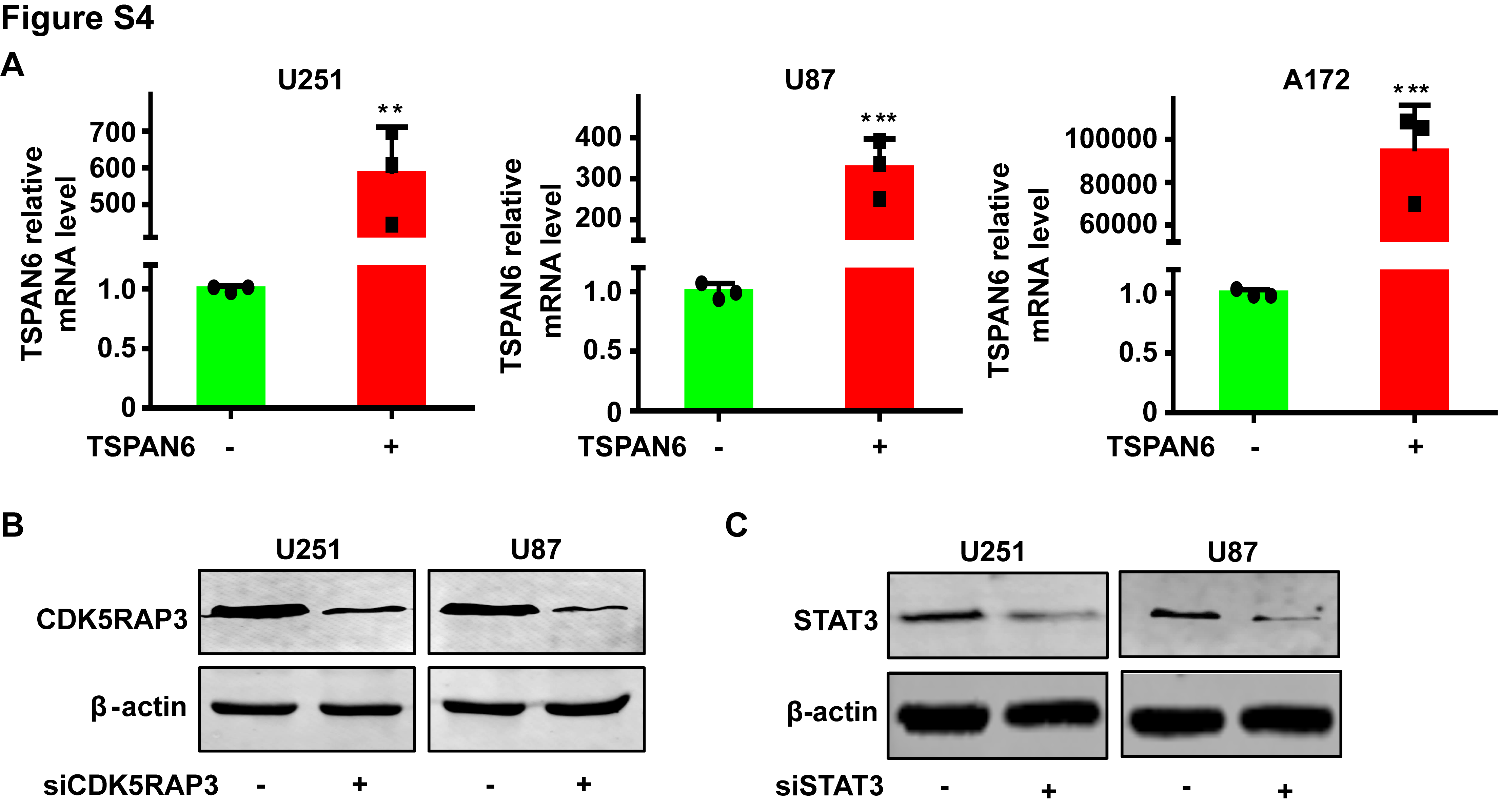

Supplement: Supplementary file 1 — Supplementary figures and tables. [file ijbsv20p2440s1.zip › Supplementary figure 4(1).tif]

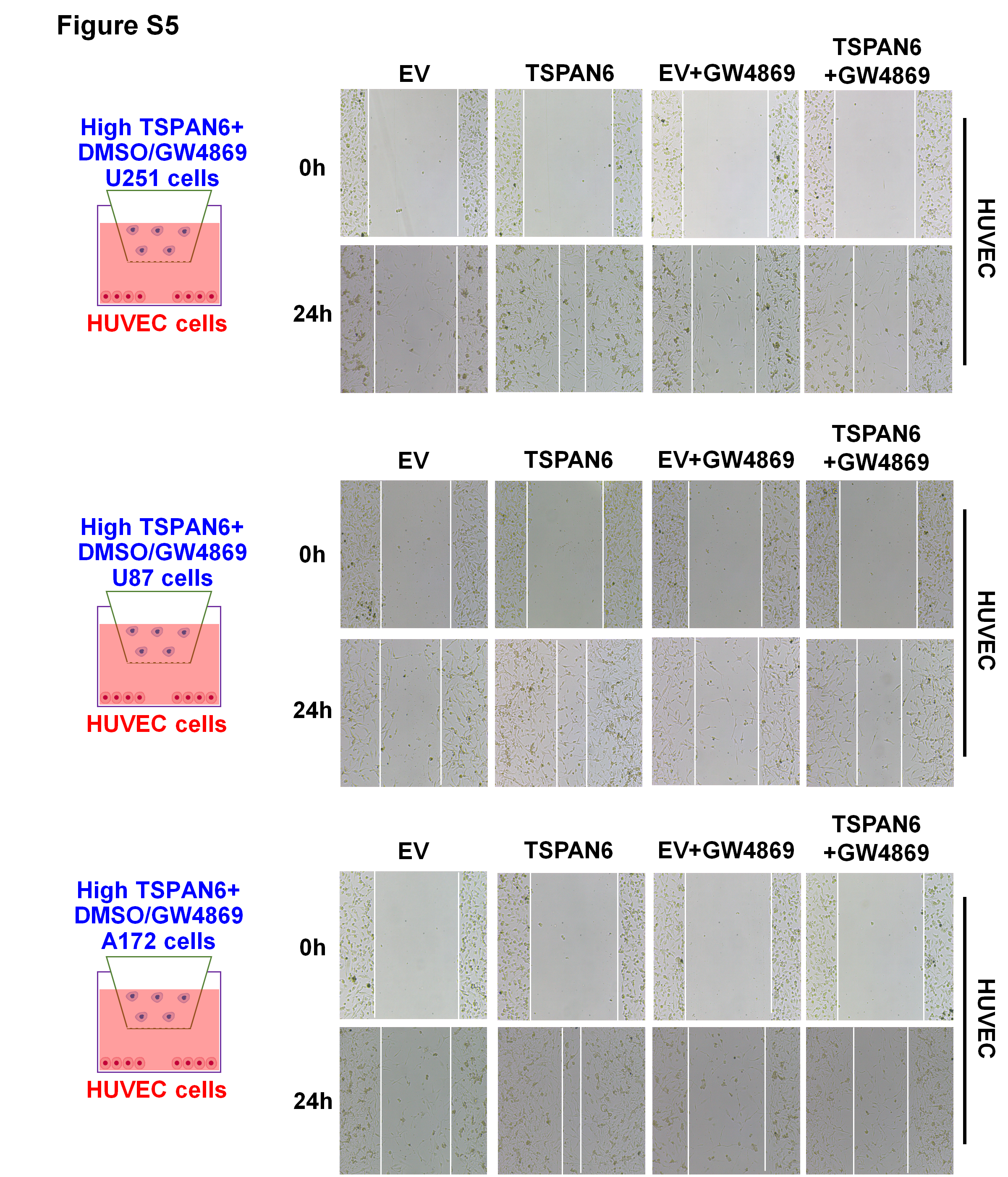

Supplement: Supplementary file 1 — Supplementary figures and tables. [file ijbsv20p2440s1.zip › Figure S5.tif]
